# Outcomes after bone marrow versus peripheral blood haploidentical hematopoietic cell transplantation using post-transplant cyclophosphamide-based graft-versus-host disease prophylaxis

**DOI:** 10.1016/j.htct.2025.106222

**Published:** 2025-11-27

**Authors:** Muhammad Kashif Amin, Moazzam Shahzad, Abat Khan, Valiko Begiashvili, Ushna Khan, Matthew McGuirk, Shaun DeJarnette, Sibgha Gull Chaudhary, Iqra Anwar, Nausheen Ahmed, Al-Ola Abdallah, Sunil H. Abhyankar, Joseph P. McGuirk, Anurag K. Singh, Muhammad Umair Mushtaq

**Affiliations:** a Division of Hematologic Malignancies & Cellular Therapeutics, University of Kansas Medical Center, Kansas City, KS, USA; b Mikael Rayaan Foundation Global Transplantation and Cellular Therapy Consortium, Kansas City, KS, USA; c H Lee Moffitt Cancer Center, Tampa, FL, USA

**Keywords:** Haploidentical hematopoietic stem cell transplant, Peripheral blood stem cell graft, Bone marrow graft, Post-transplant cyclophosphamide-based GVHD prophylaxis, Outcomes

## Abstract

**Background:**

This study aims to compare the outcomes of bone marrow (BM) to peripheral blood stem cells (PBSC) grafts in haploidentical hematopoietic cell transplantation using post-transplant cyclophosphamide-based graft-versus-host disease (GvHD) prophylaxis.

**Methods:**

A single-center retrospective analysis of all adult patients who underwent haploidentical transplants with at least one year of follow-up was conducted. Bivariate analyses were performed using chi-square tests and *t*-tests. Data were analyzed using SPSS with statistical significance being defined at p-value <0.05.

**Results:**

The study included 176 transplant recipients: 65 % received PBSC and 35 % received BM grafts. After a median follow-up of 21 months (range: 0–73 months), neither median overall survival nor disease-free survival had been reached. One-year overall survival (BM 75 % versus PBSC 74 %; p-value = 0.898) and one-year disease-free survival (63 % both groups; p-value = 0.994) were similar between groups. PBSC recipients exhibited earlier neutrophil engraftment (17 days versus 18 days; p-value = 0.022). The incidence of cytokine release syndrome was higher in PBSC (90 % versus 37 %) grafts (p-value <0.001). The incidences of Grade II-IV acute GvHD, relapse, non-relapse mortality, platelet engraftment, one-year chronic GvHD, and GvHD-free relapse-free survival were similar across both groups.

**Conclusions:**

Haploidentical HSCT recipients observed similar outcomes regardless of graft source. Marginally faster neutrophil engraftment was observed in PBSC recipients. These findings suggest flexibility in using graft source for haploidentical transplants, though prospective studies are needed to confirm these results.

Allogeneic hematopoietic stem cell transplantation (allo-HSCT) serves as a crucial therapeutic approach for a range of hematologic malignancies and non-malignant disorders [1]. However, its success relies upon the availability of suitable human leukocyte antigen (HLA)-matched donors, a challenge aggravated by donor scarcity [2]. Haploidentical stem cell transplant (haplo-HSCT) donation, when combined with post-transplant cyclophosphamide (PT-Cy), has revolutionized transplant accessibility for patients without fully matched donors [3]. This approach has confirmed the feasibility of using partially matched-related donors, significantly expanding available donor options. It demonstrates low rates of severe graft-versus-host disease (GvHD) which are comparable to those seen when bone marrow (BM) is used instead of peripheral blood stem cells (PBSC) in haplo-HSCT [4]. Additionally, high-dose PT-Cy selectively removes alloreactive T cells, effectively reducing the risk of GvHD while preserving the graft-versus-leukemia effect [5,6]. This method has particularly broadened transplant accessibility in regions with limited donor availability [[7], [8], [9]].

Published studies report that survival rates are comparable between the two graft sources, with PBSCs demonstrating lower relapse rates and faster engraftment [10,11]. However, PBSC grafts are associated with increased transplant-related mortality, primarily due to a higher incidence of GvHD [12]. In this study, the outcomes of haplo-HSCT using PT-Cy-based GvHD prophylaxis were analyzed, comparing the results based on the graft source. The aim was to clarify the relative efficacy and safety profiles of each graft source and to explore their clinical implications, thereby enhancing transplant practices and optimizing patient outcomes in haplo-HSCT.

## Methods

### Design, setting, and patients

A single-center retrospective study was conducted at the University of KS Medical Center, examining all adult haplo-HSCT recipients from August 2016 to July 2021. The study included 176 patients with at least one year of post-transplantation follow-up. The cohort consisted of adult patients who received their first allo-HSCT from donors mismatched at two or more HLA loci, independent of conditioning regimen and indication for allo-HSCT. Patients who received manipulated grafts (ex vivo or in vivo T-cell depletion, or ex vivo engineered T cells) were excluded as were those with matched unrelated, mismatched unrelated, matched related, or umbilical cord blood donors. All recipients of haplo-HSCT were administered standard GvHD prophylaxis consisting of PT-Cy, mycophenolate mofetil (MMF), and tacrolimus (continuing until Day +60 after the transplant). The myeloablative and reduced-intensity conditioning (RIC) regimens utilized are listed in Table 1. The institutional review board approved the study.

**Table 1:** Transplant conditioning regimens.

Regimen	n
Myeloablative (n = 55)	
Flu/TBI	49
Bu/Flu/Cy	6
Reduced intensity (n = 121)	
Flu/Cy/TBI	105
Flu/Mel/TBI	9
Flu/Cy/TBI/ATG	6
Flu/Cy/TBI/ATG/Thio	1

Bu: Busulfan; Cy: Cyclophosphamide; Flu: Fludarabine; TBI: Total body irradiation; Mel: Melphalan; ATG: Anti-thymocyte Globulin; Thio: Thiotepa

### Data collection, outcomes, and key definitions

Data were collected by review of the electronic medical records. Demographic, clinical, and pathologic factors were ascertained at the time of HSCT. The primary objective of this study was to compare overall survival (OS) between the groups. The secondary objectives were to compare rates of acute and chronic GvHD, cytokine release syndrome (CRS), neutrophil and platelet engraftment, non-relapse mortality (NRM), relapse, and disease-free survival (DFS). Neutrophil recovery was defined as achieving an absolute neutrophil count (ANC) >0.5 × 10^9^/L for three consecutive days. Platelet recovery was defined as achieving a platelet count >20 × 10^9^/L without transfusion requirement for seven consecutive days. Disease relapse, progression, and death were treated as events. NRM was defined as time to death without relapse or progression. Relapse was defined as the molecular, cytogenetic, or hematologic recurrence of the primary disorder. DFS was defined as survival without relapse or progression. Acute GvHD (aGvHD) was staged and graded according to the Mount Sinai aGvHD International Consortium criteria [13]. CRS was graded based on American Society for Transplantation and Cellular Therapy Consensus Guidelines [14]. Chronic GvHD (cGvHD) was staged and graded according to the 2014 National Institutes of Health criteria. Causes of death were coded according to the Center for International Blood and Marrow Transplant Research recommendations; specifically, if a patient had an active or uncontrolled GvHD and concurrent infection at the time of death, then GvHD was coded as the primary cause of death and infection was coded as a contributing cause.

### Statistical analysis

Descriptive statistics were used to compare baseline demographic characteristics. Categorical data were compared using the Chi-square test. Continuous data were compared using ANOVA or *t*-test. Univariate and multivariate Cox regression analyses were conducted to investigate factors associated with OS, DFS, NRM, and relapse including the graft source; the hazard ratio (HR) with 95 % confidence intervals (95 % CIs) were obtained. For regression models, PBSC recipients were compared with reference to BM graft recipients. Univariate regression analyses included correlation of key variables with the post-transplant outcomes, including age, sex, ethnicity, Karnofsky performance status (KPS), hematopoietic stem cell transplantation-specific comorbidity index (HSCT-CI), hematologic diagnosis, disease status (complete remission versus others), recipient cytomegalovirus (CMV) serostatus, donor CMV serostatus, donor age, donor sex, conditioning regimen, and GvHD prophylaxis. Significant factors identified in univariate analyses were entered into a multivariate analysis for the respective outcome. Data were analyzed using SPSS version 28 with statistical significance being defined as a p-value >0.05.

## Results

### Baseline and clinical characteristics

The study encompassed 176 haplo-HSCT involving 114 (65 %) PBSC and 62 (35 %) BM grafts. The median age of recipients was 54 years (range: 18–74); 119 (68 %) were male. The racial distribution was primarily Caucasian (123; 70 %), followed by Hispanic (23; 13 %), Afro-American (16; 9 %), and other ethnicities (14; 8 %). The most common hematologic diagnoses were acute myeloid leukemia (AML) in 74 patients (42 %), other myeloid disorders in 41 (23 %), acute lymphoid leukemia (ALL) in 17 (10 %), lymphoma in 33 (19 %), and other conditions in 11 (6 %). A pre-transplant HSCT-CI score of 3 or higher was recorded in 93 patients (53 %). KPS was ≥90 % in 59 (34 %) and 60–80 % in 117 (67 %) patients. RIC was administered to 121 recipients (69 %) and myeloablative conditioning (MAC) to 55 (31 %). CMV seropositivity was identified in 109 recipients (62 %) and 101 donors (57 %). At the time of transplantation, 111 recipients (63 %) were in complete remission. The median CD34^+^ cell dose was 5.0 × 10^6^ per kg for PBSC recipients and 2.8 × 10^6^ per kg for BM recipients (p-value <0.001). These data are summarized in Table 2.

**Table 2:** Patient and transplant-related characteristics of haploidentical transplants.

	Total (n = 176)	PBSC (n = 114)	BM (n = 62)	P-value
Age - median years (range)	54 (18–74)	53 (19–73)	54 (18–74)	0.706
Sex - n ( %)				
Male	119 (68)	81 (71)	38 (61)	0.238
Female	57 (32)	33 (29)	24 (39)	
Ethnicity - n ( %)				
Caucasian	123 (70)	76(67)	47(76)	0.561
African American	23 (13)	17 (15)	6 (10)	
Hispanic	16 (9)	12 (10)	4 (6)	
Others	14 (8)	9 (8)	5 (8)	5
Karnofsky performance status - n ( %)				
≥90 %	59 (33.5)	34 (30)	25 (40)	0.138
60–80 %	117 (66.5)	80 (70)	37 (60)	
Hematopoietic stem cell transplantation-specific comorbidity index - n ( %)				
0–2	83 (47)	55 (48)	28 (45)	0.753
≥3	93 (53)	59 (52)	34 (55)	
Hematologic Diagnosis - n ( %)				
Acute myeloid leukemia	74 (42)	43 (38)	31 (50)	0.027
Myeloid disorders^a^	41 (23)	33 (29)	8 (13)	
Acute lymphoblastic leukemia	17 (10)	10 (9)	7 (11)	
Lymphoma	33 (19)	24 (21)	9 (15)	
Others	11 (6)	4 (3)	7 (11)	
Disease Status - n ( %)				
Complete remission	111 (63)	63 (55)	48 (77)	0.005
Others^b^	65 (37)	51 (45)	14 (23)	
Recipient cytomegalovirus serostatus - n ( %)				
Negative	67 (38)	48 (42)	19 (31)	0.147
Positive	109 (62)	66 (58)	43 (69)	
Donor cytomegalovirus serostatus - n ( %)				
Negative	75 (43)	46 (40)	29 (47)	0.429
Positive	101 (57)	68 (60)	33 (53)	
Donor age - median years (range)	32 (10–65)	34 (11–65)	31 (10–64)	
Donor sex - n ( %)				
Male	111 (63)	73 (64)	38 (61)	0.746
Female	65 (37)	41 (36)	24 (39)	
Conditioning - n ( %)				
Myeloablative	55 (31)	47 (41)	8 (13)	<0.001
Reduced intensity conditioning	121 (69)	67 (59)	54 (87)	
Graft cell dose (Median CD34 cells x10^6^ per kg)	4.9 (4.3–5.0)	5.0 (5.0–5.2)	2.8 (2.3–3.2)	<0.001

a myeloid disorders include myelodysplastic syndromes, myeloproliferative neoplasms, and chronic myeloid leukemia.

b Includes partial response (n = 36; 8 %), stable disease (n = 89; 20 %), progressive disease (n = 31; 7 %), and not available (n = 14; 3 %).

### Primary and secondary outcomes

With a median follow-up of 21 months (range: 0–73 months), OS and DFS were not reached in either the BM or PBSC haplo-HSCT groups. The one-year OS rates were 75 % for BM and 74 % for PBSC (p-value = 0.898), and the one-year DFS rates were identical at 63 % for both groups (p-value = 0.994). PBSC recipients experienced earlier neutrophil engraftment at 17 days compared to 18 days for BM recipients (p-value = 0.022).

The incidences of Grade II-IV acute GvHD were nearly identical at 50 % for BM and 51 % for PBSC (p-value = 0.875). Relapse rates were 22 % for PBSC and 26 % for BM (p-value = 0.579), while NRM rates were 17 % for PBSC and 20 % for BM (p-value = 0.682). The median times to platelet engraftment were 28 days for PBSC and 31 days for BM (p-value = 0.092). Neutrophil recovery by Day +28 was 95 % for PBSC versus 90 % for BM (p-value = 0.349), and platelet recovery by Day +100 was 90 % for PBSC compared to 81 % for BM (p-value = 0.099). Rates of primary graft failure (PGF) and one-year chronic GvHD were also similar, with PGF at 2 % for PBSC versus 5 % for BM (p-value = 0.347) and chronic GvHD at 35 % for PBSC versus 38 % for BM (p-value = 0.410). The one-year GvHD-free relapse-free survival rate (GRFS) was 67 % for PBSC and 61 % for BM (p-value = 1.00). The overall incidence of CRS was 71 %, significantly higher in PBSC at 90 % compared to 37 % for the BM group (p-value <0.001). CRS grades were distributed as follows: Grade 1 occurred in 74 % of PBSC recipients versus 34 % for BM (p-value <0.001), Grade 2 in 14 % for PBSC and 0 % for BM (p-value <0.001), and Grade 3 and Grade 4 were both seen in 1–2 % for both groups. In the subgroup analysis stratified by conditioning intensity, neutrophil engraftment (17 days versus 18 days; p-value = 0.017) was faster with PBSC compared to BM grafts and no statistically significant association was noted in rates of acute or chronic GvHD, NRM, relapse, DFS or OS among the myeloablative transplant recipients (*n* = 55). Among the RIC transplant recipients, no statistically significant differences were noted in neutrophil and platelet engraftment, acute GvHD, NRM, Relapse, DFS, and OS between the BM and PBSC groups (Table 3).

**Table 3:** Outcomes after haploidentical transplants.

	Total (n = 176)	PBSC (n = 114)	BM (n = 62)	p-value
Follow-up - median months (range)	21 (0.3–73)	21 (0.3–69)	22 (0.3–73)	0.331
Neutrophil engraftment - median days (95 % CI)	17 (17–18)	17 (17–19)	18 (18–20)	0.022
Day +28 neutrophil recovery (>0.5 × 10^3^/µL) - n ( %)	164 (93)	108 (95)	56 (90)	0.349
Platelet engraftment - median days (95 % CI)				
>20 × 10^3^/µL	27 (26–28)	27 (26–28)	28 (26–30)	0.195
>50 × 10^3^/µL	29 (27–31)	28 (27–31)	31 (29–36)	0.092
Day +100 platelet recovery (>20 × 10^3^/µL) - n ( %)	153 (87)	103 (90)	50 (81)	0.099
Primary graft failure - n ( %)	5 (3)	2 (2)	3 (5)	0.347
Day +100 acute GvHD - n ( %)				
Grade 2–4	88 (50)	58 (51)	30 (48)	0.875
Grade 3–4	14 (8)	9 (8)	5 (8)	1.000
One-year chronic GvHD - n ( %)				
All	62 (35)	43 (38)	19 (31)	0.410
Extensive	56 (32)	38 (33)	18 (29)	0.614
Relapse - n ( %)	41 (23)	25 (22)	16 (26)	0.579
Non-relapse mortality - n ( %)	40 (23)			0.998
One-year non-relapse mortality	31 (18)	19 (17)	12 (20)	0.682
Cytokine Release Syndrome - n ( %)	125 (71)	102 (90)	23 (37)	<0.001
Cytokine Release Syndrome grade - n ( %)				
Grade 0 (No CRS)	51 (29)	12 (10)	39 (63)	<0.001
Grade 1	105 (60)	84 (74)	21 (34)	
Grade 2	16 (9)	16 (14)	0 (0)	
Grade 3	2 (1)	1(1)	1 (1.5)	
Grade 4	2 (1)	1(1)	1 (1.5)	
Cause of death - n ( %)				
Relapse/progression	22 (32)	13 (30)	9 (38)	0.599
GvHD	10 (15)	5 (11)	5 (21)	
Infections	23 (34)	17 (39)	6 (25)	
Organ failure	3 (4)	2 (5)	1 (4)	
Graft failure	3 (4)	1 (2)	2 (8)	
Others, non-transplant-related	5 (7)	4 (9)	1 (4)	
Not available	2 (3)	2 (5)	0 (0)	
Disease-free survival				
Median months (range)	NR	NR	NR	0.994
One-year disease-free survival - n ( %)	111 (63)	72 (63)	39 (63)	1.000
Overall survival				
Median months (range)	NR	NR	NR	0.898
One-year overall survival - n ( %)	131 (74)	85 (75)	46 (74)	1.000
One-year GvHD-free Relapse-free survival - n ( %)	117 (66.5)	76 (67)	41 (61)	1.000

BM: Bone marrow; PBSC: Peripheral blood stem cells; NRM: Non-relapse mortality; GvHD: Graft-versus-host disease.

## Discussion

This retrospective single-center study analyzes the outcomes of haploidentical allo-HSCT using either PBSC or BM as the graft source, with PT-Cy for GvHD prophylaxis. The study findings show comparable one-year OS and DFS rates between the two graft sources. This aligns with several other studies that have also found no significant differences in OS, DFS, and NRM between different graft sources [7,15,16].

The choice of conditioning regimen is a critical factor in haplo-HSCT and may interact with graft source to influence outcomes. In the current cohort, RIC was more frequently used in the BM group (87 % versus 59 % in PBSC; p-value <0.001), reflecting its common application in older and comorbid patients. Subgroup analysis (Table 2) revealed faster neutrophil engraftment with PBSC in MAC recipients (17 versus 18 days; p-value = 0.017), but no significant differences in acute or chronic GvHD, relapse, NRM, DFS, or OS were observed between graft sources in either the MAC or RIC cohorts. Prior studies suggest RIC may mitigate GvHD risk using PBSC grafts by reducing inflammatory responses, though potentially at the cost of higher relapse rates in certain malignancies [[15], [16], [17]]. Conversely, MAC may increase GvHD risk with PBSC due to higher CD34^+^ cell doses (median 5.0 versus 2.8 × 10^6^/kg in the present study; p-value <0.001). The imbalance in conditioning regimens limits direct comparisons, and further studies are needed to elucidate how conditioning intensity modulates graft source effects.

A notable imbalance in disease status was observed, with 77 % of BM recipients in complete remission (CR) at transplant compared to 55 % of PBSC recipients (p-value = 0.005). This disparity could bias outcomes toward the BM group, as CR is a strong predictor of improved OS and DFS. To address this, multivariate Cox regression analyses adjusted for disease status, conditioning intensity, and other covariates were performed, revealing no independent effect of graft source on OS (HR: 1.02; 95 % CI: 0.68–1.53; p-value = 0.898), DFS (HR: 1.00; 95 % CI: 0.67–1.49; p-value = 0.994), relapse (HR: 0.88; 95 % CI: 0.47–1.65; p-value = 0.579), or NRM (HR: 0.92; 95 % CI: 0.49–1.74; p-value = 0.682). These findings suggest that while CR status and conditioning are critical prognostic factors, they do not significantly alter the comparative effectiveness of PBSC versus BM in the current cohort. Nonetheless, these factors should be considered when selecting graft sources to optimize patient outcomes.

While some studies have reported better OS with PBSC (due to higher doses of CD34^+^ cells potentially leading to improved outcomes) [18], others have indicated poorer outcomes associated with higher NRM rates in PBSC recipients compared to those receiving BM grafts [8,18,19]. For example, Nagler et al. noted worse life expectancy, OS, and GRFS with PBSC [7,9,20]. The results of this study highlight the importance of considering multiple independent factors, including pre-transplant disease status, the Hematopoietic Cell Transplantation-Specific Comorbidity Index, and conditioning regimen, beyond the choice of graft source, which can significantly impact transplant outcomes [8,15,16,20].

Recipients of PBSC exhibited an earlier neutrophil engraftment by one day, indicating a potential advantage in the speed of hematopoietic recovery with PBSC grafts. However, this modest difference may not be clinically relevant. Several other studies also reported earlier neutrophil engraftment in patients who receive PBSC versus BM grafts [9,16,21]. Kato et al. demonstrated an association of total CD34^+^ dose to engraftment regardless of graft source [21]. The results here differ from other studies where similar median times for neutrophil and platelet engraftment were noted [8,18,22].

In line with previous literature [8,11,[22], [23], [24], [25]], similar incidences of Grade II-IV acute GvHD were observed between BM and PBSC recipients in this study. Consistent with previous studies, similar incidences of one-year chronic GvHD were also observed [8,16]. The incidence of GRFS, a composite outcome of GvHD and RFS, was comparable in both groups. Several other studies reported higher incidences of acute and chronic GvHD in the PBSC compared to BM group [9,15,16,18,21,22]. Various other pre-transplant factors such as low body mass index (BMI) and older age, are independently associated with a higher risk of GvHD [26,27]. No difference in graft failure was found between both cohorts in the present study. In a large retrospective study, Olsson et al. reported lower rates of PGF in patients receiving PBSC compared to BM [28]. They attributed this improvement to the higher doses of graft cells typically administered in PBSC transplants. Another factor contributing to PGF reported in literature is donor-specific anti-HLA antibodies [29,30].

CRS after haplo-HSCT is often associated with early T-cell reconstitution and low incidence of post-transplant relapse [31]. However, research has noted an increased risk of infections following CRS, potentially due to its impact on neutrophil recovery [32]. In this study, the majority of CRS cases were of mild severity (Grades 1–2), aligning with findings from previous research [33]. Additionally, the higher incidence of CRS observed in the PBSC group is consistent with earlier studies [34]. In the present study population, infections and relapse/progression of the disease emerged as the most common causes of death, consistent with findings in previous literature where GvHD-related death also featured prominently [9,22,35].

This study has several limitations inherent to its retrospective design, including selection bias, confounding by indication, and limited statistical power. With a sample size of 176 patients, the analysis may be underpowered to detect small but clinically meaningful differences in outcomes such as GvHD, relapse, or GRFS, particularly in subgroups defined by conditioning intensity or disease status. The heterogeneous patient population, encompassing diverse hematologic malignancies, ethnicities, and conditioning regimens, further complicates the generalizability of the findings. Additionally, the relatively short median follow-up of 21 months may not capture late events.

## Conclusion

These findings suggest that haplo-HSCT with PT-Cy yields comparable rates of acute and chronic GvHD, relapse, NRM, DFS, and OS between BM and PBSC graft sources. A slightly faster neutrophil engraftment with PBSC offers a potential advantage, though its clinical significance is limited. Imbalances in disease status and conditioning intensity highlight the importance of patient-specific factors in graft selection. These results suggest flexibility in choosing either BM or PBSC, allowing clinicians to tailor decisions based on donor availability and patient characteristics, such as CR status and conditioning regimen. Prospective randomized trials are needed to confirm these findings and optimize transplant strategies.

## Conflicts of interest

No relevant conflicts of interest. MH has consultancy roles in Incyte Corporation, ADC Therapeutics, Pharmacyclics, Omeros, Genmab, Morphosys, Kadmon, Kite, Novartis, Abbvie, Legend, Gamida Cell, SeaGen, Autolus, Byondis, and Forte Biosciences Inc., and is on the speaker’s bureau for Sanofi Genzyme, AstraZeneca, BeiGene, and ADC Therapeutics. JPM has consulting and advisory roles in Bristol Myers Squibb, Kite, Novartis, AlloVir, Envision, Autolus, Nektar Therapeutics, CRISPR therapeutics, Caribou Bio, Sana Technologies, Legend Biotech, and Cargo Therapeutics. MUM has research funding from Iovance Biotherapeutics. The remaining authors declare no other commercial or financial relationships that could be construed as a potential conflict of interest.
